# Postoperative Vomiting Following Laparoscopic Cholecystectomy Is Associated with Intraoperative Fluid Administration: A Retrospective Cohort Study

**DOI:** 10.3390/ijerph18105305

**Published:** 2021-05-17

**Authors:** Chia-Yu Hsieh, Yan-Yuen Poon, Ting-Yu Ke, Min-Hsien Chiang, Yan-Yi Li, Peng-Neng Tsai, Shao-Chun Wu

**Affiliations:** Department of Anesthesiology, Kaohsiung Chang Gung Memorial Hospital and Chang Gung University College of Medicine, Kaohsiung City 833, Taiwan; hsieh.yu6@gmail.com (C.-Y.H.); elephant423@gmail.com (Y.-Y.P.); vig529@hotmail.com (T.-Y.K.); ducky0421@gmail.com (M.-H.C.); yanyi6325@gmail.com (Y.-Y.L.); a0575@cgmh.org.tw (P.-N.T.)

**Keywords:** general anesthesia, laparoscopic cholecystectomy, nausea, sevoflurane, vomiting

## Abstract

Potential risk factors for postoperative vomiting (POV) are important for daily anesthesia practice. To identify the risk factors associated with POV we retrospectively reviewed 553 adult patients who underwent scheduled simple laparoscopic cholecystectomy under sevoflurane-based general anesthesia between January and December 2018. Patients who experienced POV were predominantly women, had lower body weight, and higher ASA (American Society of Anesthesiologists) physical status. The POV group showed female sex predominance, lower body weight, and higher ASA physical status, with a significant difference when compared with the non-POV group. In univariate analysis, female sex and Apfel scores of 2, 3, and 4 were associated with a higher POV incidence. Age > 70 years, higher body weight, and ASA physical status III were associated with a lower POV incidence. In multivariate logistic regression, sex, age, Apfel score, and intraoperative crystalloid infusion rate were POV predictive factors. Receiver operating characteristic analysis showed a negative association between the intraoperative crystalloid infusion rate and POV occurrence with an area under the curve of 0.73 (*p* = 0.001). The cutoff intraoperative crystalloid infusion rate was 2 mL/kg/h with 82% sensitivity and 49% specificity (≥2 mL/kg/h was associated with a lower POV incidence vs. <2 mL/kg/h (OR, 95% CI; 0.52 [0.33–0.83])). To decrease POV in these patients, identifying high-risk factors and an intraoperative crystalloid administration of ≥2 mL/kg/h should be considered in patients undergoing LC under sevoflurane-based general anesthesia.

## 1. Introduction

Postoperative nausea and vomiting (PONV) is an unpleasant experience for patients who have undergone surgery. In addition to discomfort and increased difficulty in performing activities of daily living, PONV may lead to additional undesirable complications, including dehydration, electrolyte imbalance, aspiration pneumonitis, wound dehiscence, bleeding, and esophageal rupture [1]. In addition, PONV may prolong postanesthesia care unit (PACU) stay, elevate unanticipated admission rates, and increase the costs of medical care. The incidence of PONV is approximately 30% [2], and this value is up to 80% in high-risk patients [3]. In past investigations, PONV predictors were mainly divided into patient-, anesthetic-, and surgery-related factors. Patient-related factors included sex, age, history of PONV, history of motion sickness, history of migraine, body mass index, and the patient’s physical status [4]. The Apfel scoring system has been established as a reliable tool for identifying the risk of PONV in individual patients [3]. Anesthetic factors such as duration, volatile anesthetic, and opioid use have been found to affect PONV [4]. Surgical factors, particularly cholecystectomy and laparoscopic and gynecological surgery, seem to increase the PONV rate [4]. Therefore, identifying high-risk patients, adjusting the type of anesthesia and prescribing antiemetics for PONV prevention are important issues for anesthesiologists in daily clinical practice.

Several studies have contributed to the investigation of PONV. A study revealed that patients who underwent laparoscopic cholecystectomy (LC), during which pneumoperitoneum is believed to increase the vagal impulse, exhibited a higher PONV rate than patients that underwent other types of surgeries [5]. Assessing PONV is based on a four-point severity scale ranging from “no symptoms, mild nausea, severe nausea or up to two vomits, and more than two vomits.” Because nausea is subjective and hard to measure, we used only postoperative vomiting (POV) as an endpoint in this study. We aimed to identify the risk factors for POV by retrospectively analyzing the database of a medical center in southern Taiwan. Only patients undergoing LC under sevoflurane-based general anesthesia were included in the analysis. 

## 2. Methods

This study was approved by the Institutional Review Board of Chang Gung Medical Foundation (No. 202001105B0) and was performed in accordance with the standards of the Committee on Human Experimentation. The requirement for informed consent was waived because of the retrospective study design. From January 2018 to December 2018, adult patients who underwent scheduled LC under sevoflurane-based general anesthesia in Kaohsiung Chang Gung Memorial Hospital were retrospectively reviewed to assess the potential risk factors for POV. Their medical and anesthesia records were retrieved from the electronic data base of the hospital. Patients’ medical history, demographic data, and anesthesia records were carefully reviewed and relevant data were extracted. Demographic and clinical data included age, sex, actual body weight (kg), sevoflurane consumption (mL/h), intraoperative opioid consumption, PACU stay, and stay in the ordinary ward, bispectral index (BIS) use, American Society of Anesthesiologists (ASA) physical status, anesthesia time (hour), Apfel score, prophylactic antiemetic agent use, intraoperative crystalloid administered (mL/kg/h), intraoperative urine output (mL/kg/h), intraoperative antihypertensive use, patient-controlled analgesia use, and the occurrence of POV. 

Sevoflurane consumption was automatically recorded using an Avance anesthesia machine (GE Datex-Ohmeda, Madison, WI, USA), S/5 ADU (GE Datex-Ohmeda, Madison, WI, USA), Carestation 620 (GE Datex-Ohmeda, Madison, WI, USA), or Primus (Drägerwerk AG, Lübeck, Germany). The morphine equivalent formula was used to quantify various types of opioids and routes of opioid administration [6]. At the end of surgery, nurse anesthetist will complete the anesthesia summary (such as sevoflurane-mL, crystalloid-mL, urine output-mL, etc.) in our medical records under supervision of a second senior nurse anesthetists or anesthesiologist. The records of POV were obtained from our routine postoperative interview conducted by well-trained nurse anesthetists within 24 and 72 h postoperatively. Each patient’s electronic record and the record of postoperative interview were reviewed simultaneously to ensure completeness of information. The daily postoperative visit is an integral part of our quality control system. One milligram morphine equivalent (MME) is equivalent to fentanyl 10 μg, alfentanil 75 μg, pethidine 7.5 mg, nalbuphine 1 mg, codeine 10 mg, or tramadol 12 mg (PO) [6]. Dexamethasone was used for patients with Apfel Score = 1. Dexamethasone plus ondansetron were used for patients with Apfel Score ≥ 2. If POV occurred at ward, metoclopramide was used for treatment.

The chi-square or Fisher’s exact test was used to analyze categorical variables. The Kolmogorov–Smirnov test was used to assess the normality of continuous variables. Continuous variables of patients’ clinical characteristics were compared using Student’s *t*-test. Non-normally distributed data were compared using the Wilcoxon test and expressed as medians (IQR). A univariate regression model was used to identify the relationship between the occurrence of POV and each possible variable. In addition, multivariate regression models were used to determine the influence of each variable on POV. To investigate the adequate infusion rate of intraoperative crystalloid (mL/kg/h), receiver-operating-characteristic (ROC) and area-under-the-curve analyses were used to determine the best cutoff point for POV prevention based on sensitivity and specificity. Statistical analysis was performed using SPSS (version 22.0; IBM Corp., Armonk, NY, USA). Statistical significance was set at *p* < 0.05. 

## 3. Results

**Figure 1:**
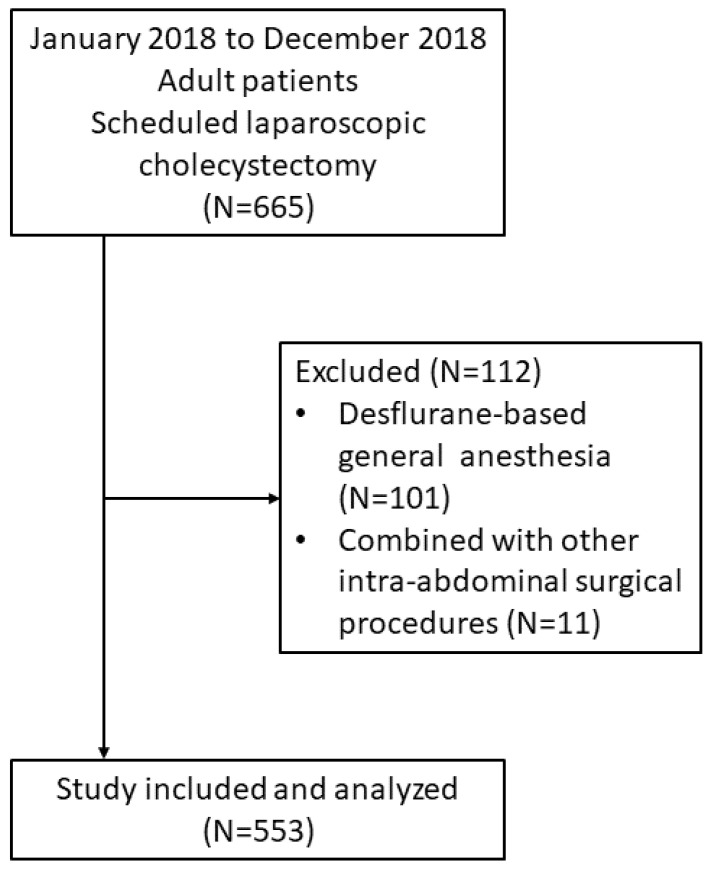
Flow diagram of the study participants.

From January 2018 to December 2018, a total of 665 scheduled laparoscopic cholecystectomies were performed under general anesthesia. A total of 553 patients who underwent scheduled LC under sevoflurane-based general anesthesia in Kaohsiung Chang Gung Memorial Hospital were included after the exclusion of 101 patients with desflurane-based general anesthesia and 11 patients with other combined intra-abdominal surgical procedures (Figure 1). 

Table 1 presents the demographic and clinical characteristic features of patients in this study. Eighty-night patients experienced POV, and 464 patients did not. Female sex predominance, lower body weight, and higher ASA physical status were found in the POV group compared to the non-POV group, with a significant difference (Table 1). There were no significant differences in age, sevoflurane consumption, morphine equivalent dose (intraoperatively/in PACU/in ordinary ward), BIS use, anesthesia time, Apfel score, types of antiemetic drugs, intraoperative crystalloid use, intraoperative urine output, antihypertensive use, and use of patient-controlled analgesia between the two groups.

**Figure 2:**
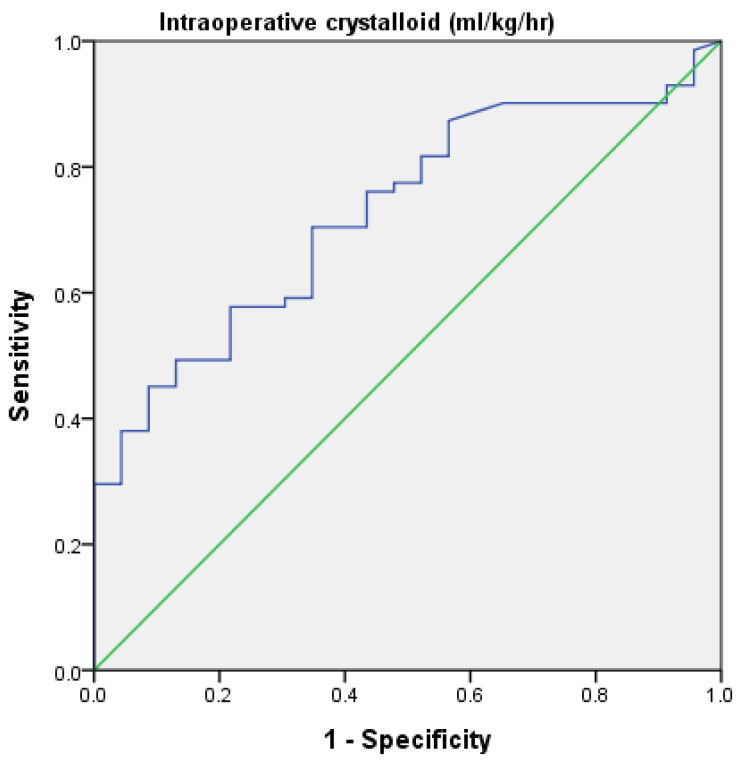
The receiver operating characteristic curve for the infusion rate of intraoperative crystalloid (mL/kg/h) for POV prediction. The area under curve was 0.727 with *p* < 0.001.

Multiple binary logistic regression analysis showed that female sex, age < 70 years, Apfel score 2, and less intraoperative crystalloid were independent factors for POV (Table 2). It is a general practice in our department that intraoperative fluid supply is maintained at a rate of 1–2 mL/kg/h in LC surgery. While in case of systolic blood pressure drops more than 25% from its baseline, a bolus of 200–400 mL crystalloid would be given. Intraoperative fluid volume was regarded as the only modifiable factor in our study model. The ROC curve (Figure 2) showed a significant negative association between the infusion rate of intraoperative crystalloids and the occurrence of POV. The area under the curve was 0.73 (*p* = 0.001). The cutoff point for the infusion rate of intraoperative crystalloid was 2 mL/kg/h, with 82% sensitivity and 49% specificity. There was a lower incidence of POV in patients with a high infusion rate (≥2 mL/kg/h) than in patients with a low infusion rate (<2 mL/kg/h) (OR (95% CI) 0.52 (0.33–0.83)).

## 4. Discussion

Our study showed female gender is an independent risk factor for POV, and it is in accordance with previous studies. A systematic review conducted by Apfel et al. showed that the strongest patient-specific predictor was the female sex (odds ratio (OR) 2.57; 95% CI (2.32–2.84)) [1]. A recent retrospective study also reported that early PONV and rescue analgesics and antiemetics were more common in women after LC [7]. 

Intraoperative fluid supply has been considered as a contributing factor for PONV. Our results supported that less intraoperative fluid supply was strongly related to POV and our results further suggested that when intraoperative fluid supply ≥2 mL/kg/h, the risk of POV was 0.52 times as in those with intraoperative fluid supply <2 mL/kg/h (OR (95% CI) 0.52 (0.33–0.83)). Intravenous fluid supplementation during general anesthesia leads to balanced electrolytes, nutrition, and intravenous fluid volume. Liberal fluid administration can lead to fluid overload after recovery from general anesthesia, delayed wound healing, and prolonged hospitalization [8]. Restrictive fluid therapy could lead to intraoperative hypotension, postoperative renal insufficiency, and organ hypoperfusion. The impact of intraoperative fluid therapy on PONV remains inconclusive. A large number of studies in a 10-year retrospective study in orthognathic surgery in Asia revealed that the more the intraoperative intravenous fluids administered, the higher the occurrence of PONV [9]. A systematic review and meta-analysis of 41 studies from the Cochrane Database showed that perioperative intravenous crystalloid supplementation could reduce PONV [10]. A randomized controlled trial showed that the incidence of POV reduced after LC in patients with a 40 mL/kg intraoperative administration of lactated Ringer’s solution than in those administered 15 mL/kg of the same solution [11]. A prospective study in gynecological laparoscopy also indicated that intravenous sodium lactate 30 mL/kg was associated with a lower incidence of PONV and antiemetic rescue when compared with sodium lactate of 15 mL/kg [12]. Our results supported the Cochrane review [10], showing that an increase in intraoperative crystalloid administration would decrease the occurrence of POV (OR 0.71, 95% CI (0.51–0.99) in the multivariate logistic regression analysis). It is reasonable to speculate that preoperative restrictions on fluid and food intake, together with bowel preparation often cause significant dehydration that may exacerbate POV. Intraoperative replenishment of the water deficit to correct hypovolemia may reduce PONV. Furthermore, our study suggests that for POV, the adequate infusion rate of intraoperative crystalloid should be ≥2 mL/kg/h.

Compared with total intravenous anesthesia with propofol, volatile anesthetics are associated with an increased incidence of PONV [13]. Volatile anesthetics are the main causes of early POV [13]. To clarify the impact of sevoflurane in our study, the total consumption of volatile anesthetics was recorded; this information was collected automatically from the anesthesia machine. There was no significant difference in the consumption of sevoflurane between the POV and non-POV groups. In addition, the consumption of sevoflurane did not affect POV on univariate and multivariate logistic regression analyses. As LC is a less traumatic surgery as compared with the open cholecystectomy, a lower consumption of sevoflurane LC would be enough to maintain a general anesthesia. This may explain sevoflurane was not a contributing factor for POV in LC.

Various opioids were prescribed in this study, including intravenous fentanyl, intravenous alfentanil, intravenous morphine, intravenous pethidine, oral tramadol, and oral tramadol-acetaminophen. The consumption of these opioids were converted to a unified unit, milligram morphine equivalent to make comparison with less bias. Mauermann et al. reported that the intraoperative fentanyl dose had a positive relationship with PONV in a prospective cohort study [14]. In another prospective study conducted by Roberts et al., opioids showed a strong logarithmic dose–response relationship with PONV postoperatively [15]. A dose–response relationship could also exist even with opioid conversion between different types and routes of administration [15]. It is generally recognized that high dose opioid is a triggering factor for nausea and vomiting [16]. However, the consumption of opioids was not a contributing factor in this study, it further supported that LC does not associated with intense surgical trauma [17], so the consumption of either intraoperative or postoperative opioid consumption is comparatively less. 

There were some limitations to our study. First, the study simply classified patients receiving elective LC under sevoflurane-based general anesthesia to control for two main confounding factors—surgical types and volatile anesthetics. Clinicians should be cautious about different surgical procedures or anesthesia techniques. It has been reported that [18] there is insufficient evidence to conclude that one general anesthetic regimen for day-procedure laparoscopic cholecystectomy is to be preferred over another. However, recent reports [19,20] showed that spinal anesthesia is preferable to general anesthesia for laparoscopic cholecystectomy, lower postoperative pain and lower PONV occurrence in spinal anesthesia. A recently published study [21] showed that patients received combined spinal-general anesthesia for laparoscopic gynecological surgery have a lower perioperative opioid consumption and a lower occurrence of PONV as compared with patients who received general anesthesia only. Second, the severity of POV was not evaluated in the present study. Third, conversion of different analgesics into a unified unit suffers from an unavoidable bias, because they have different pharmacokinetic and pharmacodynamic profiles. Finally, this was a retrospective study; studies with a higher level of evidence, such as double-blind prospective controlled trials and systematic reviews with meta-analyses, should be considered to verify our results.

## 5. Conclusions

In this study, female sex, age < 70 years, higher Apfel score, and less intraoperative fluid were independent risk factors of POV in scheduled simple LC under sevoflurane-based general anesthesia in both univariate and multivariate logistic regression analyses. It is worth noting that female sex was a strong independent risk factor for POV with an OR of 4.18 in the univariate analysis and an OR of 9.71 in the multivariate regression analysis. The adequate infusion rate for intraoperative crystalloid was ≥2 mL/kg/h for reducing POV. In contrast to previous studies, opioid use and the morphine-equivalent dose were not independent risk factors for POV. As PONV or POV involves many different pathological pathways, multimodal approach with combination of pharmacological and nonpharmacological prophylaxis along with interventions is generally employed in patients with high PONV risk to reduce baseline risk [22]. Although many new antiemetic agents have been introduced and provided considerable beneficial effects, there is still considerable lack of evidence regarding safety aspects that does warrant investigation [23]. Recently, genetic study for PONV revealed that the CHRM3 polymorphism and the Apfel score independently predict PONV susceptibility and suggested that dexamethasone/acustimulation should be considered in patients with low Apfel score but at high genetic risk [24]. An editorial [25] from Anesthesiology summarized the efforts for treating PONV over these years that “Pounds of Prevention but Only Ounces of Cure: The Need for More Research on the Treatment of Postoperative Nausea and Vomiting”. The present study otherwise suggested a feasible and effective way that could be executed in our daily practice, though more prospective studies are needed to confirm our results.

## Figures and Tables

**Table 1:** Demographic and clinical characteristic features.

Features	Total	No POV	POV	*p*-Value
Sex				
Female	296 (53.5%)	225 (48.5%)	71 (79.8%)	<0.001 ***
Male	257 (46.5%)	239 (51.5%)	18 (20.2%)	
Age (years)				
20 to 49	193 (34.9%)	155 (33.4%)	38 (42.7%)	0.087
50 to 69	250 (45.2%)	210 (45.3%)	40 (44.9%)	
70 and above	110 (19.9%)	99 (21.3%)	11 (12.4%)	
Weight (kg)	66.0 (58.0–75.5)	67.0 (59.0–77.0)	63.0 (54.5–70.5)	0.001 **
Sevoflurane consumption (mL/h)	11.3 (9.2–13.2)	11.3 (9.2–13.3)	11.5 (9.3–13.0)	0.946
Morphine equivalent: Intraoperative (mg)	13.0 (12.8–17.5)	13.0 (12.5–17.9)	13.0 (13.0–17.5)	0.539
Morphine equivalent: PACU (mg)	0.59 (0.48–0.71) ^a^	0.58 (0.45–0.70) ^a^	0.67 (0.38–0.97) ^a^	0.522
Morphine equivalent: Ward (mg)	1.42 (1.12–1.72) ^a^	1.49 (1.15–1.83) ^a^	1.09 (0.39–1.78) ^a^	0.327
BIS				
None	238 (43.0%)	207 (44.6%)	31 (34.8%)	0.088
Yes	315 (57.0%)	257 (55.4%)	58 (65.2%)	
ASA				
I	25 (4.5%)	19 (4.1%)	6 (6.7%)	0.033 *
II	392 (70.9%)	322 (69.4%)	70 (78.7%)	
III	136 (24.6%)	123 (26.5%)	13 (14.6%)	
Anesthesia time (h)				
<2	76 (13.7%)	62 (13.4%)	14 (15.7%)	0.235
2 to <4	403 (72.9%)	335 (72.2%)	68 (76.4%)	
4 and above	74 (13.4%)	67 (14.4%)	7 (7.9%)	
Types of antiemetic drugs				0.319
None	311 (56.2%)	257 (55.4%)	54 (60.7%)	
One	204 (36.9%)	172 (37.1%)	32 (35.9%)	
Two and above	38 (6.9%)	35 (7.5%)	3 (3.4%)	
Intraoperative crystalloid (mL/kg/h)	2.35 (1.80–3.05)	2.35 (1.82–3.06)	2.38 (1.79–3.02)	0.784
Intraoperative urine output (mL/kg/h)	0.38 (0.32–0.44)	0.37 (0.33–0.45)	0.31 (0.16–0.45)	0.062
Types of intraoperative antihypertensives				
None	389 (70.3%)	329 (70.9%)	60 (67.4%)	
One	137 (24.8%)	115 (24.8%)	22 (24.7%)	0.402
Two and above	27 (4.9%)	20 (4.3%)	7 (7.9%)	
Patient-controlled analgesia				
None	511 (92.4%)	426 (91.8%)	85 (95.5%)	0.228
Yes	42 (7.6%)	38 (8.2%)	4 (4.5%)	

Values are presented as N (%) or as median (IQR) unless otherwise noted. Abbreviations: IQR, interquartile range; PACU, post-anesthesia care unit; BIS, bispectral index; ASA, American Society of Anesthesiologists physical status; POV, postoperative vomiting. ^a^ expressed as mean and 95% CI. * *p*-value < 0.05. ** *p*-value < 0.01, *** *p*-value < 0.001.

**Table 2:** Univariate and multivariate logistic regression analysis of the prediction of postoperative vomiting.

	Univariate		Multivariate	
	OR (95% CI)	*p*-Value	OR (95% CI)	*p*-Value
Male	1		1	
Female	4.18 (2.42–7.25)	<0.001 ***	9.71 (2.87–33.33)	<0.001 ***
Age 20 to 49	1	0.093	1	0.096
Age 50 to 69	0.78 (0.48–1.27)	0.313	0.41 (0.16–1.04)	0.060
Age 70 and above	0.45 (0.22–0.93)	0.030 *	0.29 (0.09–0.96)	0.043 *
Body weight	0.97 (0.95–0.99)	0.002 **	0.98 (0.95–1.01)	0.104
With BIS	1		1	
Without BIS	1.51 (0.94–2.42)	0.089	1.35 (0.72–2.53)	0.351
Apfel score 0	1		1	
Apfel score 1	1.60 (0.72–3.55)	0.251	0.28 (0.08–1.03)	0.055
Apfel score 2	2.39 (1.05–5.45)	0.038*	0.14 (0.02–0.85)	0.032 *
Apfel score 3 and 4	3.30 (1.14–9.60)	0.028*	0.14 (0.02–1.06)	0.056
ASA I	1		1	
ASA II	0.69 (0.27–1.79)	0.433	0.57 (0.16–2.07)	0.389
ASA III	0.34 (0.11–0.99)	0.047 *	0.47 (0.11–2.09)	0.321
Sevoflurane consumption	0.98 (0.93–1.04)	0.551	1.04 (0.97–1.11)	0.261
Anesthesia time (hours)				
<2	1		1	
2 to <4	0.90 (0.48–1.70)	0.743	0.92 (0.40–2.11)	0.848
4 and above	0.46 (0.18–1.22)	0.120	1.10 (0.31–3.91)	0.885
Intraoperative crystalloid	0.90 (0.72–1.11)	0.310	0.71 (0.51–0.99)	0.048 *
Intraoperative urine output	0.80 (0.55–1.18)	0.261	1.09 (0.68–1.76)	0.711
Morphine equivalent: intraoperative	0.98 (0.93–1.03)	0.429	1.02 (0.95–1.10)	0.539
Morphine equivalent: PACU	1.05 (0.90–1.23)	0.540	0.99 (0.81–1.22)	0.963
Morphine equivalent: Ward	0.97 (0.90–1.04)	0.350	0.98 (0.89–1.07)	0.589
No antihypertensive	1		1	
One antihypertensive	1.05 (0.62–1.79)	0.860	1.33 (0.68–2.63)	0.405
Two or more antihypertensives	1.92 (0.78–4.74)	0.157	2.75 (0.83–9.13)	0.098
No antiemetic	1		1	
One antiemetic	0.89 (0.55–1.43)	0.618	0.59 (0.32–1.07)	0.082
Two or more antiemetics	0.41 (0.12–1.38)	0.148	0.37 (0.08–1.80)	0.218
Without PCA	1		1	
With PCA	0.53 (0.18–1.52)	0.235	0.71 (0.17–2.91)	0.633

Abbreviations: OR, odds ratio; CI, confidence interval; BIS, bispectral index; ASA, American Society of Anesthesiologists physical status; PACU, post-anesthesia care unit; PCA, patient-controlled analgesia. * *p*-value < 0.05. ** *p*-value < 0.01, *** *p*-value < 0.001.

## Data Availability

The data presented in this study are available from the corresponding authors upon reasonable request.

## References

[1] Apfel C., Heidrich F., Jukar-Rao S., Jalota L., Hornuss C., Whelan R., Zhang K., Cakmakkaya O. (2012). Evidence-based analysis of risk factors for postoperative nausea and vomiting. Br. J. Anaesth..

[2] Cohen M.M., Duncan P.G., DeBoer D.P., Tweed W.A. (1994). The postoperative interview: Assessing risk factors for nausea and vomiting. Anesth. Analg..

[3] Apfel C.C., Laara E., Koivuranta M., Greim C.A., Roewer N. (1999). A simplified risk score for predicting postoperative nausea and vomiting: Conclusions from cross-validations between two centers. Anesthesiology.

[4] Gan T.J., Belani K.G., Bergese S., Chung F., Diemunsch P., Habib A.S., Jin Z., Kovac A.L., Meyer T.A., Urman R.D. (2019). Fourth consensus guidelines for the management of postoperative nausea and vomiting. Anesth. Analg..

[5] Turgut H.C., Arslan M. (2019). An overview of treatment options for postoperative nausea and vomiting after laparoscopic surgical procedures. Anaesth. Pain Intensive Care.

[6] Back I.N. (2001). Palliative Medicine Handbook.

[7] Salazar-Parra M., Guzman-Ramirez B.G., Pintor-Belmontes K.J., Barbosa-Camacho F.J., Bernal-Hernandez A., Cruz-Neri R.U., Fuentes-Orozco C., Aguirre L.L.R., Rodriguez-Navarro D., Brancaccio-Perez I.V. (2020). Gender Differences in Postoperative Pain, Nausea and Vomiting After Elective Laparoscopic Cholecystectomy. World J. Surg..

[8] Bleier J.I., Aarons C.B. (2013). Perioperative fluid restriction. Clin. Colon. Rectal. Surg..

[9] Ghosh S., Rai K.K., Shivakumar H.R., Upasi A.P., Naik V.G., Bharat A. (2020). Incidence and risk factors for postoperative nausea and vomiting in orthognathic surgery: A 10-year retrospective study. J. Korean Assoc. Oral. Maxillofac. Surg..

[10] Jewer J.K., Wong M.J., Bird S.J., Habib A.S., Parker R., George R.B. (2019). Supplemental perioperative intravenous crystalloids for postoperative nausea and vomiting. Cochrane Database Syst. Rev..

[11] Holte K., Klarskov B., Christensen D.S., Lund C., Nielsen K.G., Bie P., Kehlet H. (2004). Liberal versus restrictive fluid administration to improve recovery after laparoscopic cholecystectomy: A randomized, double-blind study. Ann. Surg..

[12] Magner J.J., McCaul C., Carton E., Gardiner J., Buggy D. (2004). Effect of intraoperative intravenous crystalloid infusion on postoperative nausea and vomiting after gynaecological laparoscopy: Comparison of 30 and 10 mL kg(−1). Br. J. Anaesth..

[13] Apfel C.C., Kranke P., Katz M.H., Goepfert C., Papenfuss T., Rauch S., Heineck R., Greim C.A., Roewer N. (2002). Volatile anaesthetics may be the main cause of early but not delayed postoperative vomiting: A randomized controlled trial of factorial design. Br. J. Anaesth..

[14] Mauermann E., Clamer D., Ruppen W., Bandschapp O. (2019). Association between intra-operative fentanyl dosing and postoperative nausea/vomiting and pain: A prospective cohort study. Eur. J. Anaesthesiol..

[15] Roberts G.W., Bekker T.B., Carlsen H.H., Moffatt C.H., Slattery P.J., McClure A.F. (2005). Postoperative nausea and vomiting are strongly influenced by postoperative opioid use in a dose-related manner. Anesth. Analg..

[16] Coluzzi F., Pappagallo M., National Initiative on Pain Control (2005). Opioid therapy for chronic noncancer pain: Practice guidelines for initiation and maintenance of therapy. Minerva Anestesiol..

[17] Ortega A.E., Peters J.H., Incarbone R., Estrada L., Ehsan A., Kwan Y., Spencer C.J., Moore-Jeffries E., Kuchta K., Nicoloff J.T. (1996). A prospective randomized comparison of the metabolic and stress hormonal responses of laparoscopic and open cholecystectomy. J. Am. Coll. Surg..

[18] Vaughan J., Nagendran M., Cooper J., Davidson B.R., Gurusamy K.S. (2014). Anaesthetic regimens for day-procedure laparoscopic cholecystectomy. Cochrane Database Syst. Rev..

[19] Longo M.A., Cavalheiro B.T., de Oliveira Filho G.R. (2017). Laparoscopic cholecystectomy under neuraxial anesthesia compared with general anesthesia: Systematic review and meta-analyses. J. Clin. Anesth..

[20] Yu G., Wen Q., Qiu L., Bo L., Yu J. (2015). Laparoscopic cholecystectomy under spinal anaesthesia vs. general anaesthesia: A meta-analysis of randomized controlled trials. BMC Anesth..

[21] Zdravkovic M., Kamenik M. (2020). A prospective randomized controlled study of combined spinal-general anesthesia vs. general anesthesia for laparoscopic gynecological surgery: Opioid sparing properties. J. Clin. Anesth..

[22] Shaikh S.I., Nagarekha D., Hegade G., Marutheesh M. (2016). Postoperative nausea and vomiting: A simple yet complex problem. Anesth Essays Res..

[23] Weibel S., Schaefer M.S., Raj D., Rucker G., Pace N.L., Schlesinger T., Meybohm P., Kienbaum P., Eberhart L.H.J., Kranke P. (2020). Drugs for preventing postoperative nausea and vomiting in adults after general anaesthesia: An abridged Cochrane network meta-analysis(&ddagger; section sign). Anaesthesia.

[24] Klenke S., de Vries G.J., Schiefer L., Seyffert N., Bachmann H.S., Peters J., Frey U.H. (2018). CHRM3 rs2165870 polymorphism is independently associated with postoperative nausea and vomiting, but combined prophylaxis is effective. Br. J. Anaesth..

[25] Darvall J.N., Leslie K. (2019). Pounds of Prevention but Only Ounces of Cure: The Need for More Research on the Treatment of Postoperative Nausea and Vomiting. Anesthesiology.

